# Genetic Ancestry, Social Classification, and Racial Inequalities in Blood Pressure in Southeastern Puerto Rico

**DOI:** 10.1371/journal.pone.0006821

**Published:** 2009-09-09

**Authors:** Clarence C. Gravlee, Amy L. Non, Connie J. Mulligan

**Affiliations:** 1 Department of Anthropology, University of Florida, Gainesville, Florida, United States of America; 2 Genetics Institute, University of Florida, Gainesville, Florida, United States of America; University of Illinois at Champaign-Urbana, United States of America

## Abstract

**Background:**

The role of race in human genetics and biomedical research is among the most contested issues in science. Much debate centers on the relative importance of genetic versus sociocultural factors in explaining racial inequalities in health. However, few studies integrate genetic and sociocultural data to test competing explanations directly.

**Methodology/Principal Findings:**

We draw on ethnographic, epidemiologic, and genetic data collected in southeastern Puerto Rico to isolate two distinct variables for which race is often used as a proxy: genetic ancestry versus social classification. We show that *color*, an aspect of social classification based on the culturally defined meaning of race in Puerto Rico, better predicts blood pressure than does a genetic-based estimate of continental ancestry. We also find that incorporating sociocultural variables reveals a new and significant association between a candidate gene polymorphism for hypertension (α_2C_ adrenergic receptor deletion) and blood pressure.

**Conclusions/Significance:**

This study addresses the recognized need to measure both genetic and sociocultural factors in research on racial inequalities in health. Our preliminary results provide the most direct evidence to date that previously reported associations between genetic ancestry and health may be attributable to sociocultural factors related to race and racism, rather than to functional genetic differences between racially defined groups. Our results also imply that including sociocultural variables in future research may improve our ability to detect significant allele-phenotype associations. Thus, measuring sociocultural factors related to race may both empower future genetic association studies and help to clarify the biological consequences of social inequalities.

## Introduction

The current, interdisciplinary debate over racial inequalities in health has revived controversy about the role of race in health-related sciences [1]–[3]. Some researchers defend race as a useful construct for identifying the genetic basis of susceptibility to complex disease [4], [5]. Others view race as a cultural construct that corresponds poorly to genetic variation and warn that continued use of the race concept obscures the fundamental social causes of racial inequities in health [6]–[9]. This debate often moves in circles, however, because few studies integrate genetic and sociocultural data to test competing explanations for racial inequalities in health directly—despite consensus that both genetic and environmental factors are integral to complex disease [10].

Two common problems contribute to this limitation. First, race is often used uncritically as a proxy for unspecified genetic, sociocultural, or behavioral risk factors [11]–[13]. Such usage makes it impossible to untangle the independent influence of genetic and sociocultural factors or to detect interactions between them. Second, studies that do test specific genetic or sociocultural hypotheses seldom test competing explanations [14]–[16]. Consequently, causal inferences about racial inequalities in health are often confounded by unmeasured genetic or sociocultural factors associated with racial classification.

To address these limitations, we introduce a measurement strategy designed to isolate the presumed genetic and sociocultural dimensions of race. Our premise is that health researchers often implicitly use “race” in reference to two distinct concepts: genetic ancestry versus social classification. This conceptual distinction is widely recognized [16]–[18], but it has not been translated into measurement. In particular, to our knowledge, no previous study has tested the associations between genetic ancestry, social classification, and health outcomes. Here we integrate ethnographic, genetic, and epidemiologic data to fill this gap. We estimate individual genetic ancestry using 78 ancestry informative markers (AIMs), genetic variants that show large frequency differences between continental groups [19]. We assess social classification with an ethnographically grounded measure that estimates how people are perceived in everyday social interaction [20]. Last, we assay candidate gene polymorphisms for a complex phenotype, blood pressure.

We focus on the relationship between African ancestry and blood pressure [21]. This relationship is significant for two reasons. The first is its impact on public health. In the United States, hypertension is almost 50 percent more common among Black Americans than it is among Whites [22] and contributes more to racial inequalities in life expectancy than does any other cause of death [23]. A trend toward high blood pressure has also been documented among people of African ancestry in Latin America and the Caribbean [21], [24]–[26]. This pattern has deadly consequences, as hypertension and cardiovascular disease have emerged as leading causes of mortality throughout the Americas [27].

Second, the relationship between African ancestry and blood pressure is significant because it has long been a focus of debate over the relative importance of genetic versus environmental influences on racial inequalities in health. Some researchers have interpreted African ancestry—initially estimated by skin pigmentation, later by genetic markers—as a proxy for racial-genetic predisposition to disease [28]–[30] Others have proposed that higher African ancestry increases the risk of exposure to poverty, racism, and other social stressors related to hypertension [20], [31]–[33]. Debate between these alternatives has foundered, in part, because previous studies have not isolated the presumed genetic and sociocultural dimensions of race implicit in each hypothesis. Indeed, lack of clarity about the genetic and sociocultural meanings of race is a far-reaching problem [34]. Thus, our attempt to isolate African genetic ancestry from the sociocultural implications of ancestry is potentially relevant to the broader goal of explaining and eliminating racial inequalities in health.

We draw on preliminary data collected in southeastern Puerto Rico. This research setting is apt because the history of the sugar economy and slavery in this region led us to expect substantial variation in levels of African ancestry [35]. Also, previous work in Puerto Rico linked blood pressure to observer classification of skin color [36] and to social classification based on *color*—an analog to the concept of race in the United States [20]. These studies imply that social classification shapes exposure to stressors related to blood pressure, but they do not address directly the relative importance of social classification versus genetic ancestry. Previous ethnographic research suggests that Puerto Rico is a particularly valuable setting for teasing these variables apart because social classification based on *color* is shaped not only by skin color by also by other physical features and non-biological markers of social status [37], [38]. Thus, for a given level of genetic admixture, there should be variability in social classification, making it possible to isolate the presumed genetic and sociocultural dimensions of race.

We take advantage of this setting to ask three questions: (1) How is social classification based on *color* associated with individual genetic ancestry? (2) Does genetic ancestry or *color* better predict variation in arterial blood pressure? (3) Does the inclusion of sociocultural data alter the association between blood pressure and candidate gene polymorphisms for hypertension? Our results show, for the first time, that (a) social classification better predicts blood pressure variation than does genetic ancestry and (b) accounting for sociocultural aspects of race reveals a significant association between a genetic variant (α_2C_ adrenergic receptor deletion) and blood pressure. These preliminary results have important implications for future research on racial inequalities in health.

## Results

### How is color associated with individual genetic ancestry?

Our measure of *color* estimates how people are perceived by other Puerto Ricans in everyday social interaction. This estimate improves on standard observer ratings [39], because it links respondents directly to systematic ethnographic data on the culturally appropriate system of social classification in this setting (see Materials and Methods). Our respondents fell into three categories of ascribed *color*: *blanco*, or white (n = 37); *trigueño*, the major intermediate category (n = 31), and *negro*, or black (n = 19). These categories correspond to statistically significant differences in both genetic ancestry and education, but not in other study variables (Table 1). Respondents classified as *negro* have slightly elevated mean SBP, as compared to *blancos* or *trigueños*, but this bivariate association is not statistically significant.

**Table 1:** Descriptive statistics for study variables.

	Blanco (n = 37)	Trigueño (n = 31)	Negro (n = 19)	Total (N = 87)
Systolic blood pressure	124.79 (15.96)	124.44 (20.59)	128.45 (16.46)	125.46 (17.71)
Diastolic blood pressure	79.74 (10.37)	80.37 (11.05)	80.21 (12.25)	80.07 (10.91)
Age, y	40.08 (7.42)	37.52 (8.06)	39.05 (9.48)	38.94 (8.11)
Sex, % female	56.76	74.19	42.11	59.77
Body mass index	28.75 (5.30)	28.96 (6.49)	27.52 (7.11)	28.56 (6.11)
Education, y*	15.59 (2.63)	13.06 (4.55)	12.47 (2.74)	14.01 (3.69)
Household income	6.05 (2.58)	3.39 (3.07)	3.31 (2.19)	4.51 (2.98)
Antihypertensive medication, %	10.81	12.9	0.0	9.2
African ancestry*	0.19 (0.10)	0.28 (0.12)	0.44 (0.11)	0.28 (0.15)

Means (SD) are reported for continuous variables, percentages for categorical variables.

* ANOVA shows statistically significant differences across categories of *color* for education and African ancestry.

**Figure 1:**
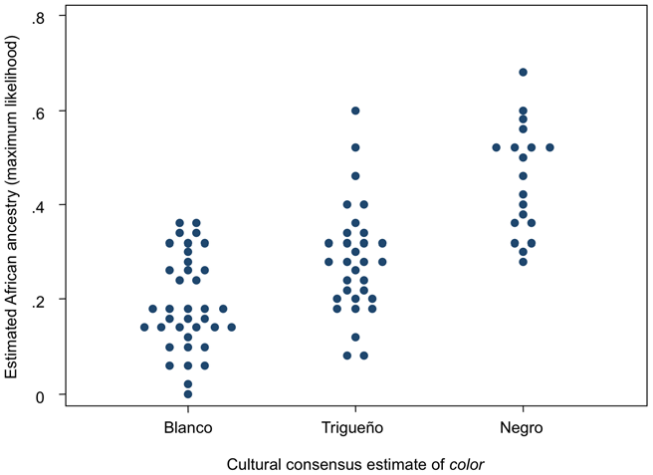
Relationship between individual level of African genetic ancestry and culturally ascribed *color*. Mean genetic ancestry varies across categories of *color* (*blanco* versus *trigueño: F*[1,84] = 12.04, *p* = .003; *blanco* versus *negro*: *F*[1,84] = 73.65, *p*<.001; *trigueño* versus *negro*: *F*[1,84] = 29.31, *p*<.001; Šidák-correction for multiple comparisons). Individual African ancestry ranges from 0.08 to 0.69 in our sample. The distribution of African ancestry overlaps for *blanco* and *trigueño* across 40 percent of the observed range (0.08–0.35); for *trigueño* and *negro* across 48 percent (0.27–0.59); and for all three *color* categories across 12 percent (0.27–0.35).

Figure 1 shows the relationship between individual African genetic ancestry and culturally ascribed *color*. There are statistically significant differences in mean genetic ancestry between all pairs of *color* categories. For our purposes, however, the overlap between categories is more important than are mean differences, because it confirms the expectation that respondents with a given level of African ancestry may vary in social classification (Figure 1). At least two *color* categories overlap across 40–48% of the observed range of estimated ancestry, and all three categories overlap across 12% of the total range. Thus, neither genetic ancestry nor *color* is a strong predictor of the other, making it necessary to measure each variable separately.

### Does genetic ancestry or color better predict variation in blood pressure?

Using multiple linear regression, we first tested the association between individual African genetic ancestry and blood pressure, controlling for age, sex, body mass index (BMI  =  weight kg/height m^2^), and use of antihypertensive medication (Tables 2 and 3, Model A). Genetic ancestry was positively associated with SBP (*p* = 0.037) but not DBP (*p* = 0.326) when adjusted only for standard covariates.

**Table 2:** Multiple linear regression coefficients (b) for systolic blood pressure (SBP) on genetic ancestry, ascribed *color*, socioeconomic status, α_2C_ adrenergic receptor genotype, and standard covariates.

	Model A		Model B		Model C		Model D	
	b	*p*	b	*p*	b	*p*	b	*p*
Constant	120.84	0.000	120.96	0.000	128.44	0.000	130.92	0.000
Age (years)	1.02	0.000	1.15	0.000	1.07	0.000	1.23	0.000
Sex (1 = male, 0 = female)	7.02	0.026	8.55	0.006	6.44	0.048	7.46	0.000
Body mass index	−0.02	0.944	0.01	0.966	−0.04	0.879	0.00	0.998
Antihypertensive use	19.53	0.001	21.87	0.000	19.90	0.001	22.42	0.000
African ancestry	22.22	0.037	18.18	0.176	27.55	0.017	19.96	0.139
Socioeconomic status			−2.00	0.294			−1.50	0.446
Ascribed *color*								
Trigueño versus Blanco			−3.44	0.348			−2.86	0.435
Negro versus Trigueño/Blanco			3.94	0.444			8.39	0.136
SES * ascribed *color*								
SES * Trigueño versus Blanco			−3.95	0.253			−4.23	0.228
SES * Negro versus Trigueño/Blanco			13.92	0.003			17.14	0.001
α_2C_Del322-325 homozygotes					−12.60	0.107	−19.47	0.007
α_2C_Del322-325 heterozygotes					−1.56	0.665	−1.58	0.619
Adjusted R^2^	0.378	0.000	0.495	0.000	0.379	0.000	0.530	0.000

Body mass index (BMI)  =  weight kg/(height m)^2^; SES  =  socioeconomic status; antihypertensive  =  self-reported use of antihypertensive medication.

**Table 3:** Multiple linear regression coefficients (B) for diastolic blood pressure (DBP) on genetic ancestry, ascribed *color*, socioeconomic status, α_2C_ adrenergic receptor genotype, and standard covariates.

	Model A		Model B		Model C		Model D	
	b	*p*	b	*p*	b	*p*	b	*p*
Constant	79.10	0.000	79.04	0.000	79.15	0.000	81.06	0.000
Age (years)	0.50	0.000	0.58	0.000	0.51	0.001	0.59	0.000
Sex (1 = male, 0 = female)	0.53	0.811	1.91	0.407	0.40	0.862	1.59	0.505
Body mass index	0.12	0.516	0.16	0.376	0.10	0.592	0.15	0.431
Antihypertensive use	8.17	0.037	9.98	0.010	8.05	0.048	10.04	0.013
African ancestry	7.37	0.326	4.62	0.645	8.03	0.325	5.41	0.608
Socioeconomic status			−2.06	0.150			−2.01	0.195
Ascribed *color*								
Trigueño versus Blanco			−2.01	0.466			−1.93	0.502
Negro versus Trigueño/Blanco			0.87	0.822			1.79	0.684
SES * ascribed *color*								
SES * Trigueño versus Blanco			0.04	0.988			0.12	0.967
SES * Negro versus Trigueño/Blanco			7.06	0.040			7.66	0.048
α_2C_Del322-325 homozygotes					−2.99	0.593	−5.58	0.315
α_2C_Del322-325 heterozygotes					0.43	0.869	0.15	0.952
Adjusted R^2^	0.172	0.001	0.249	0.000	0.150	0.007	0.232	0.002

Body mass index (BMI  =  weight kg/(height m)^2^); SES  =  socioeconomic status; antihypertensive  =  self-reported use of antihypertensive medication.

Next we added *color* and a measure of socioeconomic status (SES), as well as the interaction between *color* and SES (Tables 2 and 3, Model B); previous research had identified the interaction as associated with blood pressure [20], [31], [32], [40]. The interaction between *color* (*negro* versus *trigueño/blanco*) and SES was significantly associated with SBP (*p* = 0.003), while evidence for an association between genetic ancestry and SBP disappeared (*p* = 0.176). The interaction and simple main effects of SES and *color* account for an additional 11.7% of variance in SBP and 7.7% of variance in DBP, as compared to models without those variables. In contrast, we found no evidence of an interaction between SES and genetic ancestry. This pattern suggests that whatever exposures related to blood pressure were initially captured by the estimate of genetic ancestry are better modeled by *color* and SES.

**Figure 2:**
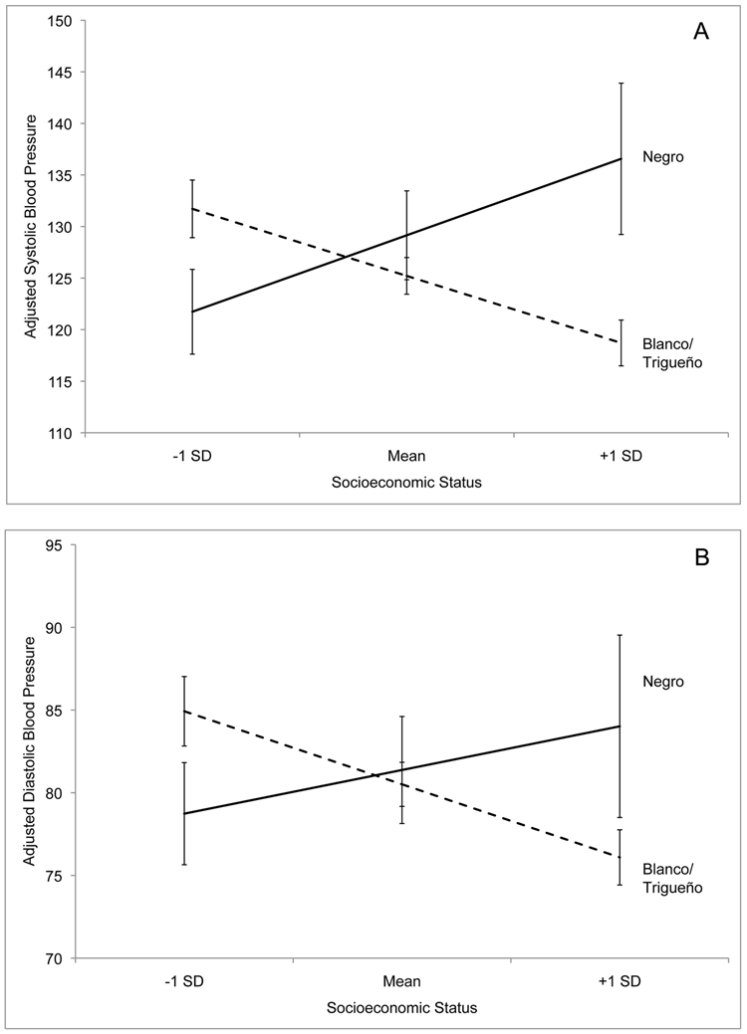
Interaction effect between culturally ascribed *color* and socioeconomic status for (A) systolic blood pressure and (B) diastolic blood pressure, adjusted for age, sex, body mass, use of antihypertensive medications, and genetic ancestry.

Figure 2 illustrates the interaction between SES and *color* in relation to SBP and DBP, adjusting for covariates. The figure facilitates interpretation because it reveals that the direction of the association between SES and blood pressure varies between categories of *color*. For respondents who are culturally defined as *blanco* or *trigueño*, SBP and DBP decrease as SES increases. In contrast, for respondents culturally defined as *negro*, SBP and DBP increase along with SES. Mean blood pressure differences between categories of *color* are greatest at high levels of SES.

### Does the inclusion of sociocultural data alter the association between blood pressure and candidate gene polymorphisms for hypertension?

Last, we tested the association between blood pressure and six candidate gene polymorphisms. Without the sociocultural data, there was no evidence of a statistically significant association between any of the candidate gene alleles and SBP or DBP (Tables 2 and 3, Model C, data shown only for α_2C_Del322-325). However, when *color* and SES were added to the model, the Del322-325 polymorphism in the α_2C_ adrenergic receptor gene showed a significant association with SBP. Specifically, α_2C_Del322-325 homozygotes showed a negative association with SBP (Table 2, Model D). A similar decrease in *p*-value was seen when sociocultural data were added to the model for DBP, although none of the DBP results were statistically significant (Table 3, Model D). The statistical significance of the result for SBP is remarkable, given the relatively small sample size. No consensus exists regarding the best method to correct for multiple testing in association studies. However, even a conservative Bonferroni correction (corrected p-value = 0.05/6 assayed polymorphisms  = 0.0083) yields a significant association between α_2C_Del322-325 homozygotes and SBP (p = 0.007).

The composition of α_2C_Del322-325 homozygotes may explain why the inclusion of sociocultural data changed the allele:phenotype association. Among α_2C_Del322-325 homozygotes, there were three categories of people: *negro*/low-SES, *negro*/high-SES, and *trigueño* + *blanco*/low-SES. Within each category, α_2C_Del322-325 homozygotes had lower SBP relative to non-deletion homozygotes (an average reduction in SBP of 14 mmHg). Specifically, mean SBP for α_2C_Del322-325 homozygotes versus non-deletion homozygotes was 136.0 versus 152.0 mmHg for the combination *negro*/high-SES, 111.5 versus 129.9 mmHg for *negro*/low-SES, and 117.6 versus 125.5 mmHg for *trigueño* + *blanco*/low-SES. Thus, *color* and SES were needed to partition the sociocultural heterogeneity within α_2C_Del322-325 homozygotes and reveal a genetic association that was otherwise hidden.

## Discussion

The role of race in health-related research is among the most controversial issues in science. Our study is significant because it answers repeated calls to measure both genetic and sociocultural factors in research on racial inequalities in health [41]–[43]. We introduce a measurement strategy that attempts to isolate two variables for which race is often used as a proxy—genetic ancestry and social classification—and we test how well each accounts for health disparities. We show that social classification, not genetic ancestry, is associated with blood pressure through an interaction with SES, and we provide the first evidence that measuring sociocultural variables reveals a genetic association that was otherwise hidden. These preliminary results suggest that measuring the sociocultural dimensions of race may both help to clarify the biological consequences of social inequalities and empower future genetic association studies. This possibility reinforces the need to integrate the social and life sciences in future research on racial inequalities in health.

### Hypertension in the African Diaspora

The relationship between African ancestry and blood pressure is a valuable test case because it has been taken as evidence of both genetic and sociocultural mechanisms. Boyle [28] first observed that darker-skinned African Americans had higher mean blood pressures than did their lighter-skinned counterparts. On the assumption that darker skin color signified greater African admixture, he interpreted this pattern as evidence of a racial-genetic predisposition for hypertension; others followed suit [29], [44]. More recently, researchers have pursued this hypothesis with genetic-based estimates of continental ancestry. Tang et al. [30] found a positive—but not statistically significant—association between blood pressure and individual African admixture in a large sample of African Americans. They interpreted the association as evidence of genetic differences between Africans and non-Africans in susceptibility to high blood pressure, although they did not include variables that would test competing hypotheses.

Other researchers argue that the apparent association between African ancestry and high blood pressure could be mediated by sociocultural factors [20], [31]–[33]. In racially stratified societies, social classification based on the perception of race shapes exposure to many environmental factors related to blood pressure, including SES, residential context, interpersonal discrimination, and structural constraints on health-related behaviors [45]–[47]. On average, darker-skinned people fare worse on these factors than do their lighter-skinned counterparts [48]. Consequently, the association between individual African ancestry and blood pressure could reflect higher exposure to adverse environmental conditions among people with greater levels of African admixture, rather than functional genetic differences related to blood pressure.

Our study is the most direct test of these alternative explanations to date. We find that social classification based on *color* is associated with blood pressure through an interaction with SES, but genetic ancestry is not. The form of the interaction between SES and *color* mirrors results from an earlier analysis of this sample using skin pigmentation as a proxy for genetic ancestry [20]. This finding is not surprising, given the moderately strong correlation between skin pigmentation and estimated African ancestry in our sample (*r* = 0.63). However, the use of a genetic-based estimate of ancestry in our current analysis provides a more direct test of competing explanations for excess hypertension in the African Diaspora. Furthermore, the analysis in our current study shows that the interaction between SES and *color* is robust to the addition of hypertension candidate gene variants.

The positive association between SES and blood pressure for respondents classified as *negro* may seem unexpected; in industrialized societies, increased household income and education are often associated with better health. However, the pattern we observe is consistent with the hypothesis that social classification based on *color* entails differential exposure to social stressors related to blood pressure. In particular, there is ethnographic evidence that Puerto Ricans perceived as *negro*, as compared to *trigueño* or *blanco*, may encounter more frequent frustrating interactions in high-SES settings due to institutional and interpersonal discrimination [38], [49]. Such interactions appear to have physiological consequences, including elevated blood pressure [50], [51]. Recent evidence suggests that the interaction between class and color may shape health inequalities in similar ways elsewhere in the African Disapora, notably in Brazil and the mainland United States [31], [32], [52], [53].

### Role of Race and Genetic Ancestry in Health Disparities Research

Our findings have implications for the role of genetic ancestry in health disparities research, beyond the specific debate over racial inequalities in blood pressure. Recent advances in genomic technology have led to the escalating use of AIMs in health-related research. The primary purpose of estimating ancestry using AIMs is to control for population structure, which, if neglected, can cause spurious associations in genetic association studies [54]. Some researchers, however, have begun to use ancestry estimates as a proxy for the presumed genetic component of racial inequalities in complex disease [30], [40], [55]–[60]. Unfortunately, this practice obscures rather than clarifies the relative importance of genetic or environmental factors and the interaction between them.

The critical limitation is that most previous studies of the relation between genetic ancestry and health neglect the sociocultural implications of genetic ancestry. If African ancestry is associated with socially defined race [61], and socially defined race shapes exposure to environmental influences on human biology [62], then studies that ignore or underestimate the implications of social classification are likely to be confounded by unmeasured environmental factors [16]. Indeed, our findings provide the most direct support yet for the hypothesis that reported associations between genetic ancestry and complex phenotypes may operate through sociocultural mechanisms. This evidence places the burden on future researchers to rule out sociocultural explanations for the association between genetic ancestry and complex phenotypes.

A related implication concerns the need to refine the measurement of racial classification as a sociocultural variable. Social scientists almost uniformly espouse the view that race is a cultural construct, but it is often expressed only to reject racial-genetic determinism, not to stimulate empirical research on *how* race is culturally constructed. Our measurement strategy is innovative, because it explicitly links survey measurement to ethnographic data on how race is culturally constructed in a particular context. This strategy is consistent with other measurement approaches, notably observer ratings [39] and the recent use of “socially assigned race” in the Behavioral Risk Factor Surveillance Survey (BRFSS) [63]. However, our approach improves on these methods by linking ethnography and survey methods to measure (1) how race is culturally constructed in a particular context and (2) how people are assigned to racial categories according to the prevailing cultural model of racial classification.

Although our findings support a sociocultural explanation for the link between African ancestry and blood pressure, they do not deny the importance of genetic influences on complex disease. On the contrary, our study provides empirical support for the theoretical expectation that improved measurement of environmental factors improves the ability to detect associations between candidate gene polymorphisms and complex phenotypes [64]. Specifically, we observed an association between the α_2C_Del322-325 variant and SBP only when *color* and SES were included in the analysis. The inclusion of *color* and SES may also explain why our results differ from previous studies of this polymorphism, which have produced conflicting results. The most recent, largest study (n = 3398) found no association with the α_2C_Del322-325 polymorphism and hypertension [65]. Our results suggest that undetected sociocultural heterogeneity may have masked significant allele-phenotype associations in previous studies. Improved measurement of relevant sociocultural variables may thus empower future genetic association studies.

### Future Directions

Our conclusions warrant additional research in several directions. First, in comparison to related studies, our sample size is small. It is noteworthy, however, that we detected statistically significant associations despite the small sample size, and that the form of the interaction effect is consistent with larger studies [32]. Second, the error in individual estimates of ancestry tends to be high, especially when using a limited number of AIMs [66]. In our sample, the average 95% confidence interval for estimated African ancestry was 0.27; the 2-unit support interval was 0.15. Future studies should assay additional AIMs to test whether improving the precision of ancestry estimates alters the pattern of findings we report here. Third, we lack data on dietary intake or physical activity. Previous studies indicate that skin color and exposure to social stressors are associated with blood pressure independent of nutritional factors [31], [36], but measuring diet and physical activity would enhance future research. Fourth, direct comparisons between estimates of African ancestry in Puerto Rico and in the mainland United States are complicated by different histories of genetic admixture. It remains for future studies to determine whether the associations we observed can be replicated in other settings, including among African Americans in the United States. Last, although our measure of social classification improves on existing approaches, further research is needed to assess how well it approximates the ascription of *color* in everyday social interaction. Future research could build on our measurement approach by testing whether non-biological markers of social status (e.g., hair style, dress, speech) influence social classification [67], whether using photographs rather than standardized portraits produces more accurate estimates, and whether the approach works as well in other cultural contexts. Additional measures of exposure to institutional and interpersonal racism would also enhance future research [68].

## Materials and Methods

### Ethics Statement

The research protocol was approved by the University of Florida Institutional Review Board. Informed consent was obtained from all participants prior to data collection.

### Research Setting and Sampling

Data were collected in Guayama, Puerto Rico, a town of approximately 44,000 people on the southeastern coast of the island [69]. Sampling procedures have been described previously [20], [70]. Briefly, the survey was conducted in four residential areas selected ethnographically to represent contrasts in two key independent variables: socioeconomic status and *color*. Within each area, we drew a simple random sample of 25 households and then randomly selected one adult, aged 25–55 years, from each household (N = 100). Ninety-six participants donated buccal swabs and gave informed consent for DNA analyses. After removing samples and markers with >25% missing data, our dataset included 87 individuals. The combination of probability and non-probability sampling methods limits the generalizability of our results but improves the efficiency of our attempt to isolate contrasts related to socioeconomic status and *color*.

### Genotyping

Samples were genotyped for 100 biallelelic autosomal SNP markers at Prevention Genetics (Marshfield, Wisconsin). These ancestry informative markers (AIMs) were selected for large frequency differences between West African, European, and Native American populations, derived from a GeneMapping 10K Affymetrix array. Eighty-seven individuals were typed for 78 AIMs. Eighty-four individuals were also assayed via pyrosequencing at the University of Florida Center for Pharmacogenomics for six hypertension candidate genes within three different genes of the adrenergic receptor family: Ser49Gly and Arg389Gly in β_1_AR, Gly16Arg, Gln27Glu, and Arg523Arg in β_2_AR, and Del322-325 in α_2C_AR.

### Genetic Ancestry and Social Classification

Three independent methods were used to estimate individual genetic ancestry: two Bayesian approaches using a Markov chain Monte Carlo algorithm, implemented in *Structure* 2.2 [71] and ADMIXMAP [72], and a maximum likelihood method [73] implemented in software provided by Xianyun Mao. Genotypes and allele frequencies from unadmixed populations of West Africans, Europeans, and Native Americans (needed for *Structure* and ADMIXMAP/MLE, respectively) were provided by Mark Shriver. Ancestral proportions for K = 3 were selected for all three programs because of the putative ancestral contribution from three distinct populations in Puerto Rico [74]. The three methods did not produce significantly different ancestry estimates and yielded similar results in our analyses; we present maximum likelihood estimates.

Our measure of *color* estimates how people are perceived by other Puerto Ricans in mundane social interaction. In an earlier ethnographic study in the same community where the survey was conducted, Gravlee [75] identified locally relevant categories of *color*. He asked ethnographic informants to identify the *color* of 72 standardized facial portraits that varied systematically in skin tone, hair texture, and facial features. He then used cultural consensus analysis [76] to test the assumption that respondents shared a coherent cultural model of *color* classification and to determine the culturally appropriate categorization of each portrait. In the current study, two observers determined which of the same 72 portraits most closely resembled each respondent, as described elsewhere [20]. We then used the consensus categorization of the matching portrait as an estimate of each respondent's ascribed *color*. This method produces an ethnographically grounded estimate of how survey respondents would be perceived by others in everyday social interaction.

### Blood Pressure and Covariates

Blood pressure was measured using an automatic oscillometric blood pressure monitor (Omron HEM-737AC; Omron Healthcare, Inc., Vernon Hills, IL) that has been validated for population-based studies [77], [78]. Three measurements were taken at standardized intervals at the beginning, middle, and end of the hour-long interview. Respondents had been seated for at least 10 minutes and had not ingested caffeine or tobacco for at least 30 minutes before each measurement. We took measurements with the left arm supported at heart level. Mean systolic (SBP) and diastolic blood pressure (DBP) from the three measurements were treated as dependent variables.

Standard covariates included sex (0 = female, 1 = male), age (years), socioeconomic status (SES), body mass index (BMI, weight in kg/height in m^2^), and current use of antihypertensive medication (0 = no, 1 = yes). Weight (0.1 kg) was measured with a digital scale; height (0.1 cm) was measured with a portable stadiometer. SES was estimated as a combination of self-reported education (years) and household income (nine categories; total from all sources, before taxes, in the last 12 months). We tested multiple ways of modeling SES [79]. Here we used scores on the first principal component of education and household income (88% common variance explained); other ways of modeling SES did not alter substantive results.

### Statistical Analysis

Regression analyses were performed separately for SBP and DBP. Ascribed *color* was entered into models as two categorical variables using a reverse Helmert coding scheme. The first variable tested for differences between *trigueño* (literally, “wheat-colored”) and *blanco* (white); the second for differences between *negro* (black) and the mean of *trigueño* and *blanco*. This coding scheme reflects both the natural ordering of categories and the expectation from ethnography that the stigmatized category *negro* would differ from the mean of *trigueño* and *blanco*.

Based on previous research [20], [31], [32], [40], we constructed cross-product terms to test for two-way interactions between (a) SES and genetic ancestry and (b) SES and *color*. Continuous predictors were mean-centered to reduce multicollinearity and to ease interpretation of interaction terms. We examined variance inflation factors for multicollinearity and case diagnostics for evidence of influential observations. In models including α_2C_AR genotype, three participants with missing genotype data for this polymorphism were excluded from the analysis. We replicated all other regression models without these three participants and observed no significant differences in any coefficients.
